# Supplementary material for: A biomimetic ocular prosthesis system: emulating autonomic pupil and corneal reflections
Source: Nat Commun. 2022 Nov 9;13:6760. doi: 10.1038/s41467-022-34448-6 (PMC9646703; doi:10.1038/s41467-022-34448-6)
Supplement: Supplementary file 1 — Supplementary Information [file 41467_2022_34448_MOESM1_ESM.pdf]

Supplementary Information

A Biomimetic Ocular Prosthesis System: Emulating Autonomic Pupil and Corneal Reflections

Seongchan Kim[1,2], Yoon Young Choi[3], Taewan Kim[4,5], Yong Min Kim[6], Dong Hae Ho[7], Young Jin Choi[8], Dong Gue Roe[9], Ju-Hee Lee[10], Joongpill Park[4], Ji-Woong Choi[11], Jeong Won Kim[11], Jin-Hong Park[1,10], Sae Byeok Jo[12], Hong Chul Moon[6,*], Sohee Jeong[4,5,*], Jeong Ho Cho[8,*]

[1]SKKU Advanced Institute of Nanotechnology (SAINT), Sungkyunkwan University, Suwon 16419, Korea.
[2]Department of Engineering Science and Mechanics, The Pennsylvania State University, University Park, PA 16802, USA.
[3]Department of Mechanical Science and Engineering, University of Illinois at Urbana Champaign, Urbana, IL 61801, USA.
[4]Department of Energy Science and Center for Artificial Atoms, Sungkyunkwan University, Suwon 16419, Korea.
[5]Sungkyun Institute of Energy Science and Technology (SIEST), Sungkyunkwan University, Suwon 16419, Republic of Korea
[6]Department of Chemical Engineering, University of Seoul, Seoul 02504, Korea.
[7]Mechanical Engineering, Soft Materials and Structures Lab, Virginia Tech, Blacksburg, VA 24061, USA.
[8]Department of Chemical and Biomolecular Engineering, Yonsei University, Seoul 03722, Korea.
[9]School of Electrical and Electronic Engineering, Yonsei University, Seoul 03722, Korea.
[10]Department of Electrical and Computer Engineering, Sungkyunkwan University, Suwon 16419, Korea
[11]Korea Research Institute of Standards and Science (KRISS), Daejeon 34113, Korea.
[12]School of Chemical Engineering, Sungkyunkwan University (SKKU), Suwon 16419, Republic of Korea.

*Corresponding author: H.C.M (hcmoon@uos.ac.kr), S.J (s.jeong@skku.edu), J.H.C (jhcho94@yonsei.ac.kr)

Table of Contents

Supplementary Fig. 1 ········ 3

Supplementary Fig. 2 ········ 3

Supplementary Fig. 3 ········ 4

Supplementary Fig. 4 ········ 4

Supplementary Fig. 5 ········ 5

Supplementary Fig. 6 ········ 5

Supplementary Fig. 7 ········ 6

Supplementary Fig. 8 ········ 6

Supplementary Fig. 9 ········ 7

Supplementary Fig. 10 ········ 8

Supplementary Fig. 11 ········ 9

Supplementary Fig. 12 ········ 9

Supplementary Fig. 13 ········ 10

Supplementary Fig. 14 ········ 10

Supplementary Fig. 15 ········ 11

Supplementary Fig. 16 ········ 11

Supplementary Fig. 17 ········ 12

Supplementary Fig. 18 ········ 12

Supplementary Fig. 19 ········ 12

Supplementary Fig. 20 ········ 13

Supplementary Fig. 21 ········ 13

Supplementary Fig. 22 ········ 14

Supplementary Fig. 23 ········ 14

Supplementary Fig. 24 ········ 15

Supplementary Fig. 25 ········ 15

Supplementary Fig. 26 ········ 15

Supplementary Table 1 ········ 16

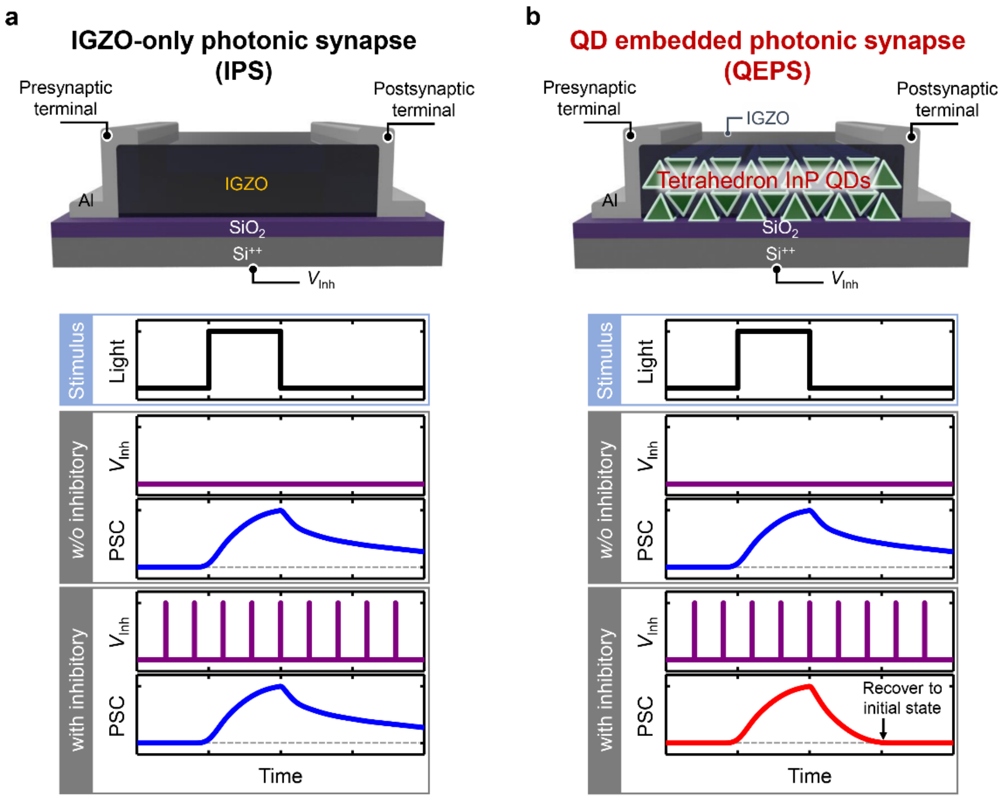

Supplementary Fig. 1. Schematic structures and signals. Comparison of the structure and LTP/D characteristics of **a** IPS and **b** QEPS.

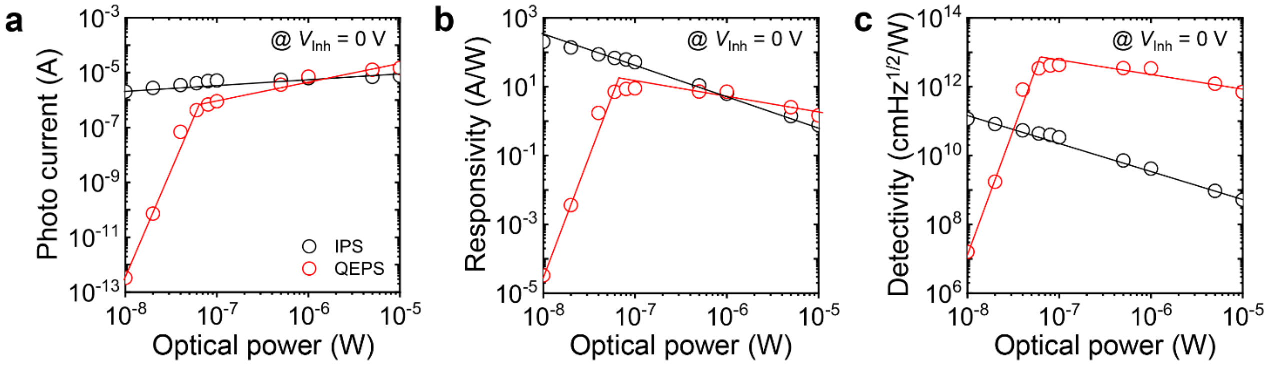

Supplementary Fig. 2. Optical performance of the QEPS. a Photo current, **b** responsivity, and **c** detectivity of photonic synapses.

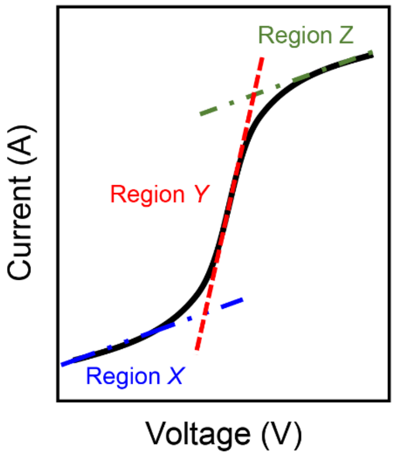

Supplementary Fig. 3. Schematic of the typical logarithmic plot of the current-voltage curve. Region *X*, region *Y*, and region *Z* correspond to Ohmic behavior, trap-limited space charge limited current, and Mott's trap-free V^2 law, respectively.

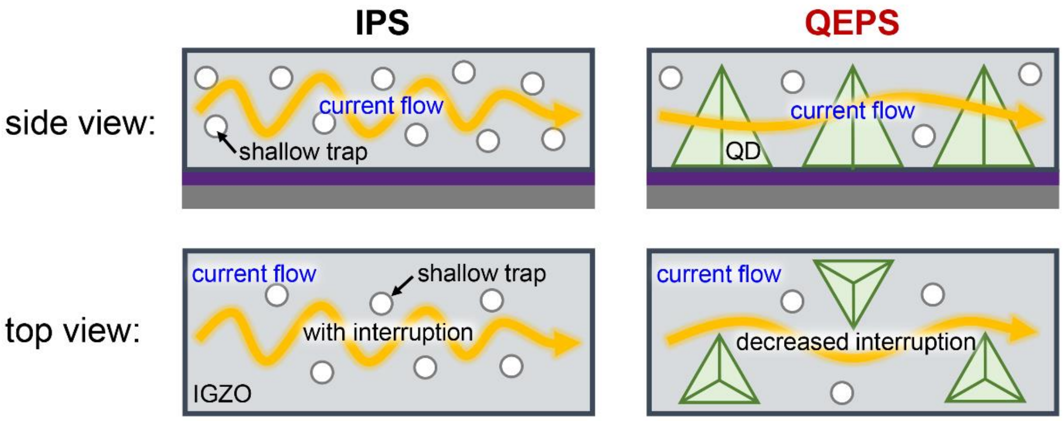

Supplementary Fig. 4. Schematic of current flow path in photonic synapses. The movement of carriers in the QEPS is less disturbed by traps which results in a higher PSC of QEPS under illumination.

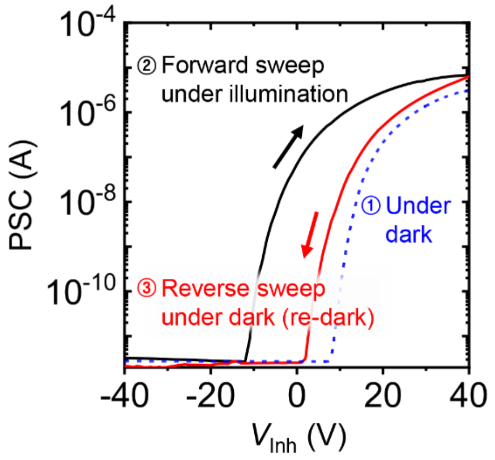

Supplementary Fig. 5. Semilogarithmic plot of transfer characteristics of QEPS in various conditions. The influence of V_{Inh} on the PSC was measured sequentially under the dark-illumination-dark (re-dark) condition

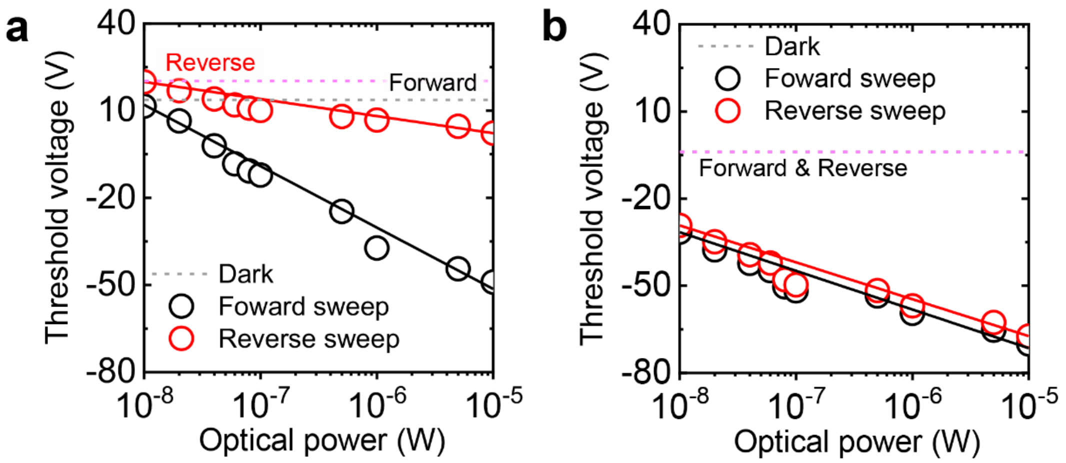

Supplementary Fig. 6. Threshold voltage of photonic synapses. a QEPS and **b** IPS under forward sweep (illumination) and reverse sweep (re-dark).

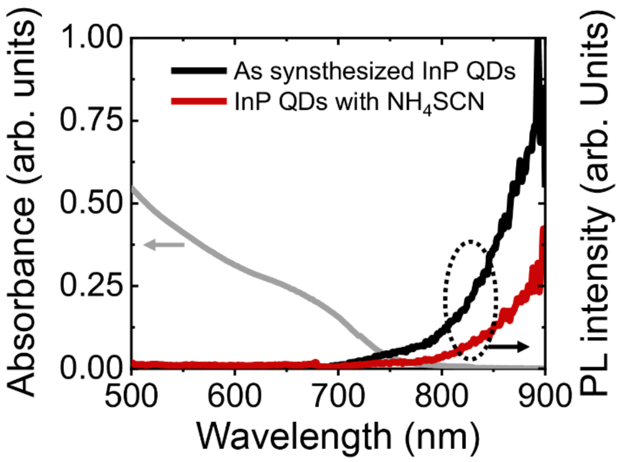

Supplementary Fig. 7. Optical properties of as-synthesized InP QDs and InP QDs with NH_4SCN ligand. Trap emission in the wavelength region above 800 nm at PL intensity indicates the presence of the mid-gap trap.

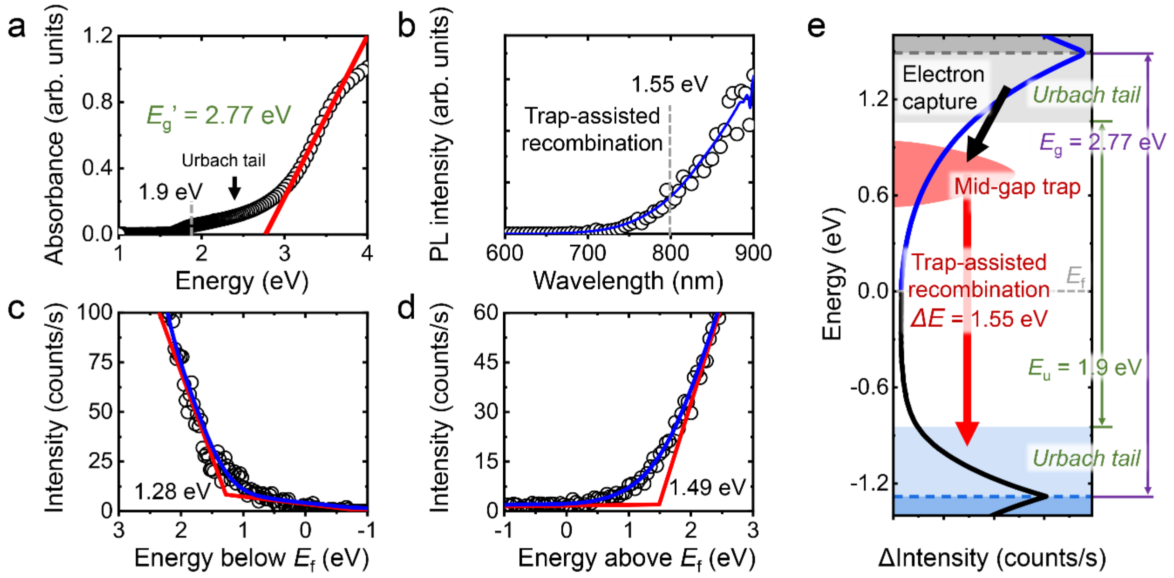

Supplementary Fig. 8. DOSs distribution of InP QDs. a light absorption, **b** PL emission spectra, **c** UPS, and **d** IPES of InP QDs. **e** Band structure of InP QDs calculated from **a**-**d**.

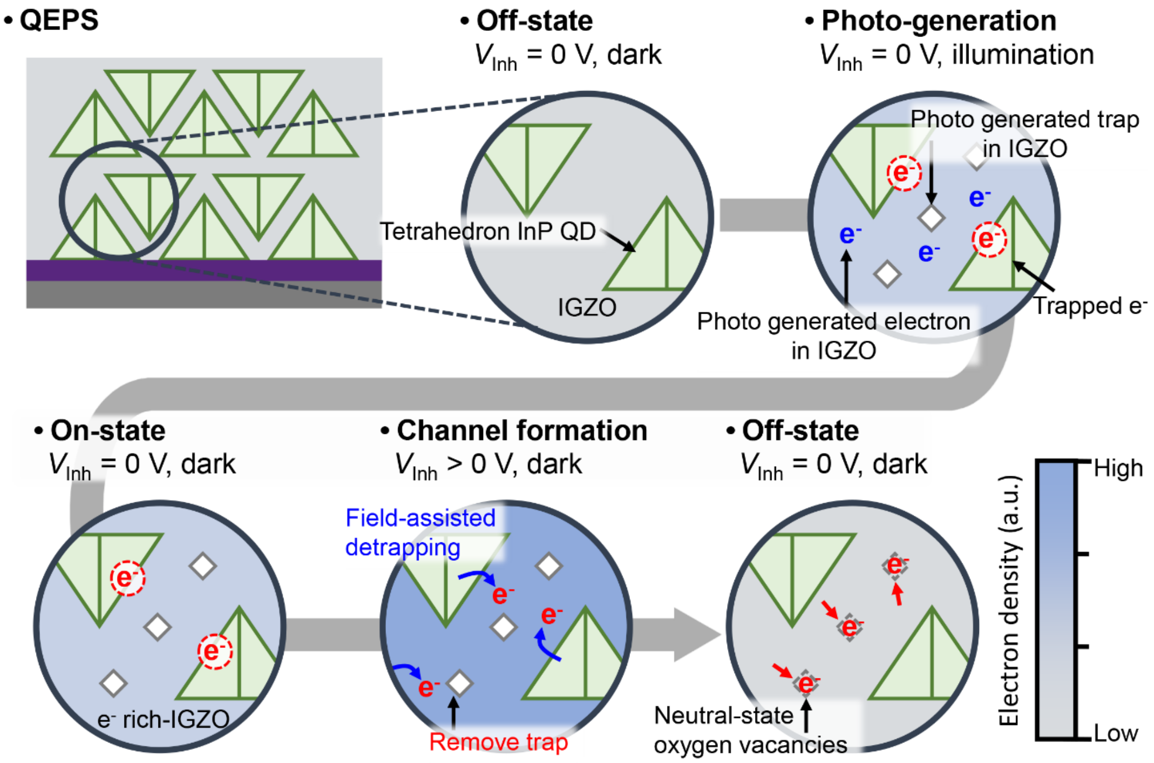

Supplementary Fig. 9. Schematic of recombination process near the gate insulator in the QEPS. This electron trapping-detrapping cycle occurs in the entire QEPS channel.

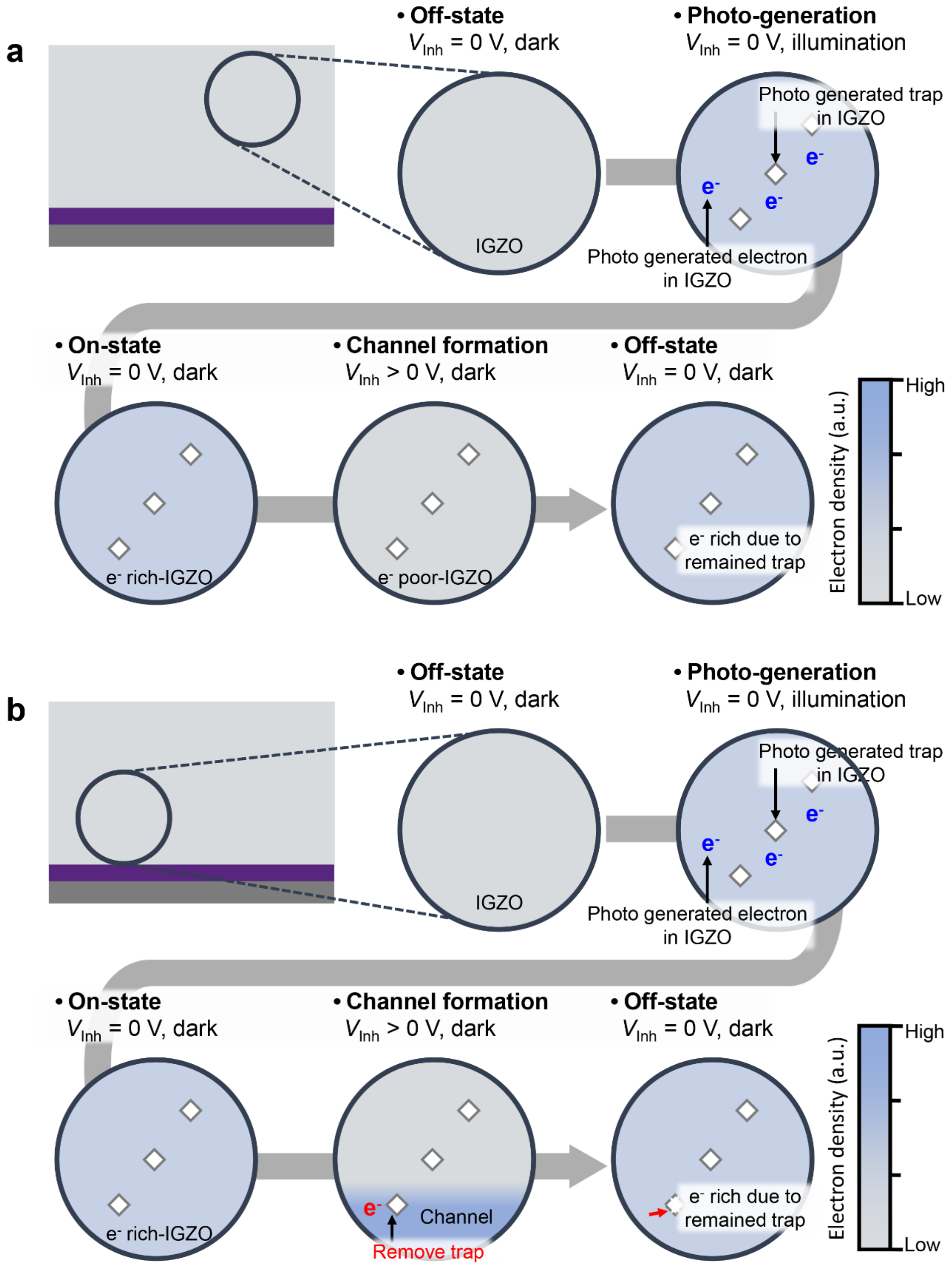

Supplementary Fig. 10. Schematic of recombination process in the IPS. a near the surface and **b** near the gate insulator.

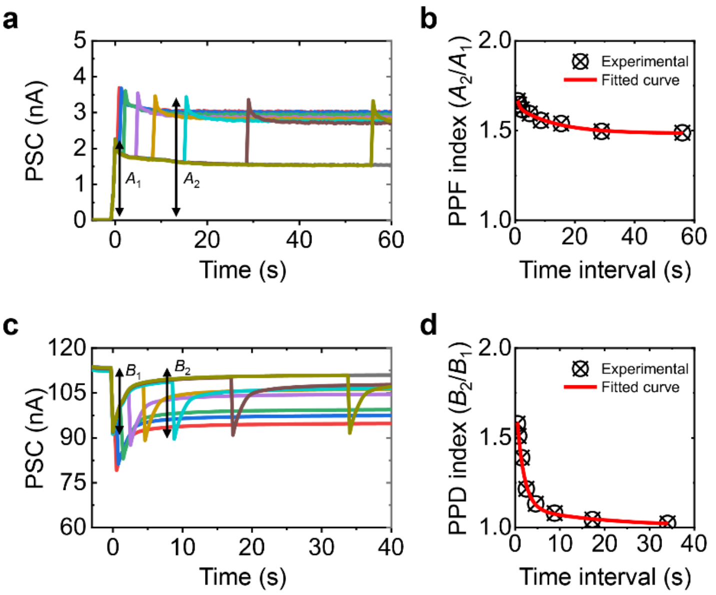

Supplementary Fig. 11. Paired-pulse behavior of the QEPS. a PSC behavior and **b** paired-pulse facilitation (PPF) index of the QEPS. **c** PSC behavior and **d** paired-pulse depression (PPD) index of the QEPS.

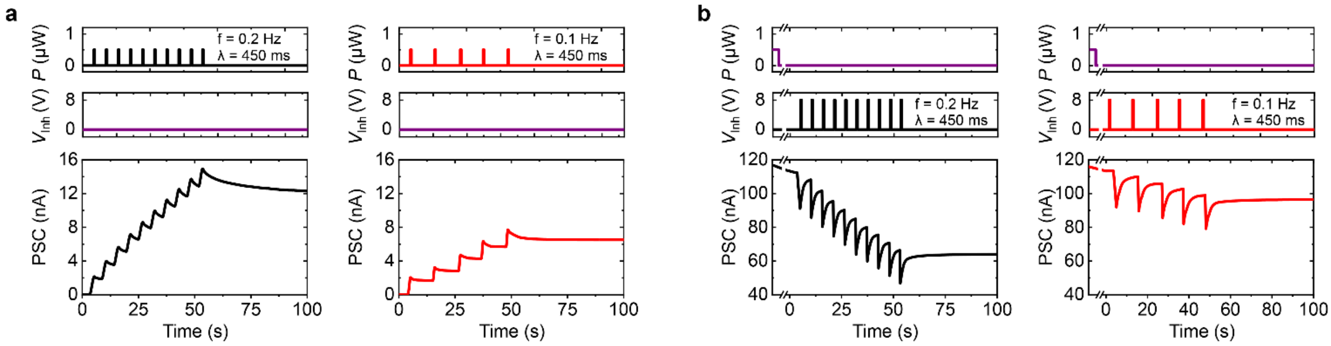

Supplementary Fig. 12. Short-term behavior of the QEPS. a short-term potentiation (0.2 Hz and 0.1 Hz for left panel and right panel, respectively) and **b** short-term depression of the QEPS (0.2 Hz and 0.1 Hz for left panel and right panel, respectively).

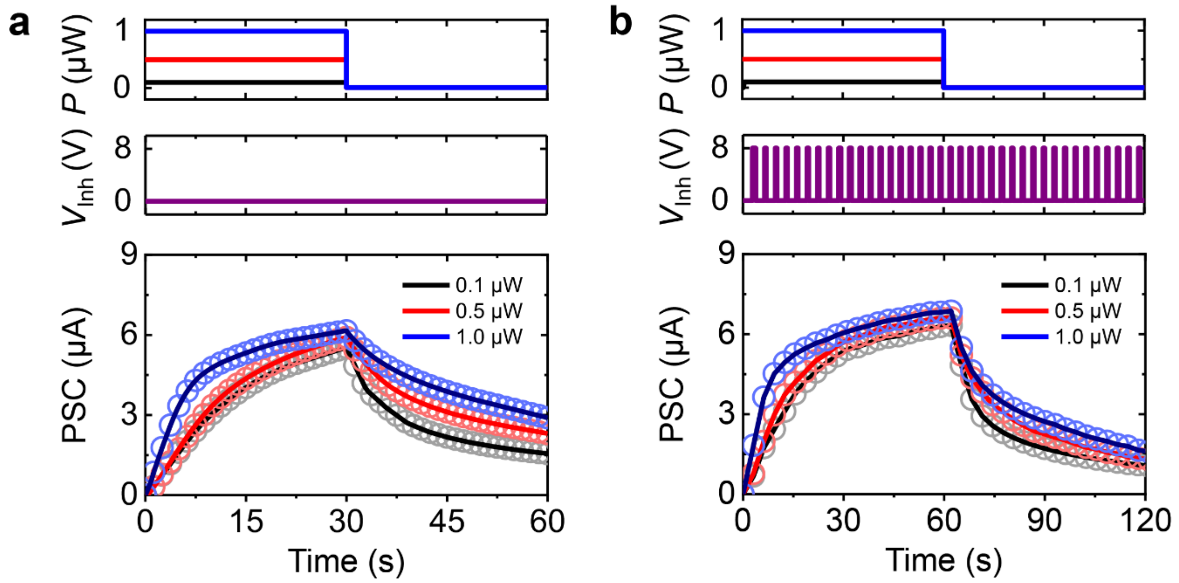

Supplementary Fig. 13. LTP/D behavior of the IPS. a without and **b** with application of V_{Inh} pulse train under various light intensities.

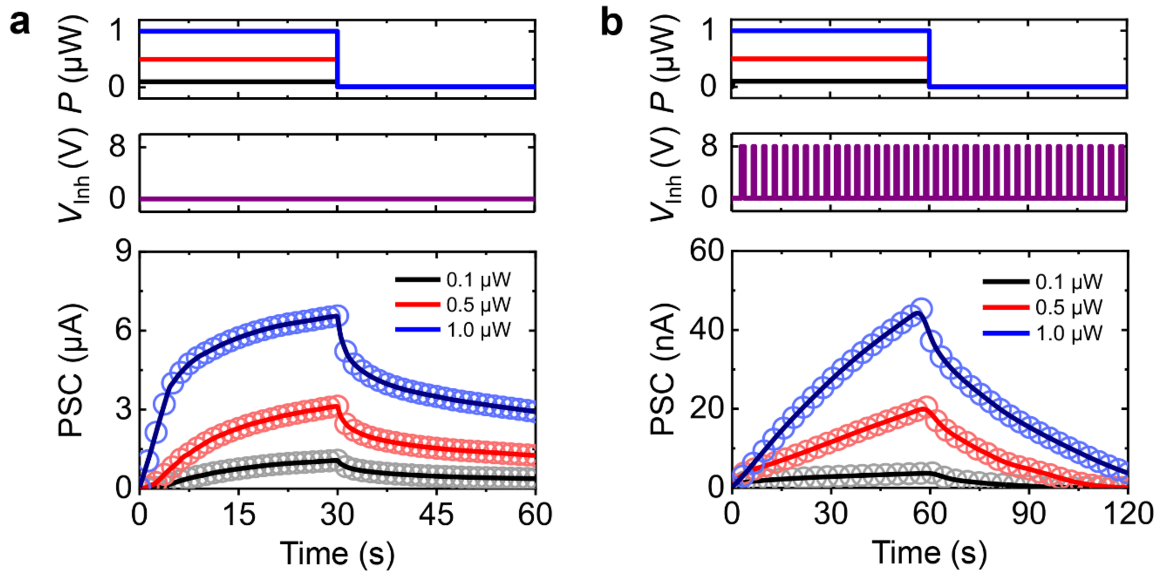

Supplementary Fig. 14. LTP/D behavior of the QEPS. a without and **b** with application of V_{Inh} pulse train under various light intensities.

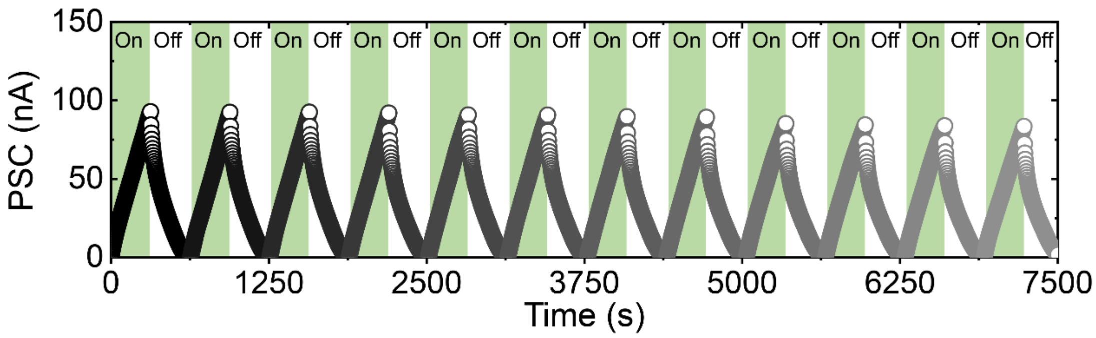

Supplementary Fig. 15. LTP/D behavior of the QEPS during the multiple cycles of illumination and dark conditions. Stable LTP/D behavior was observed for 12 cycles.

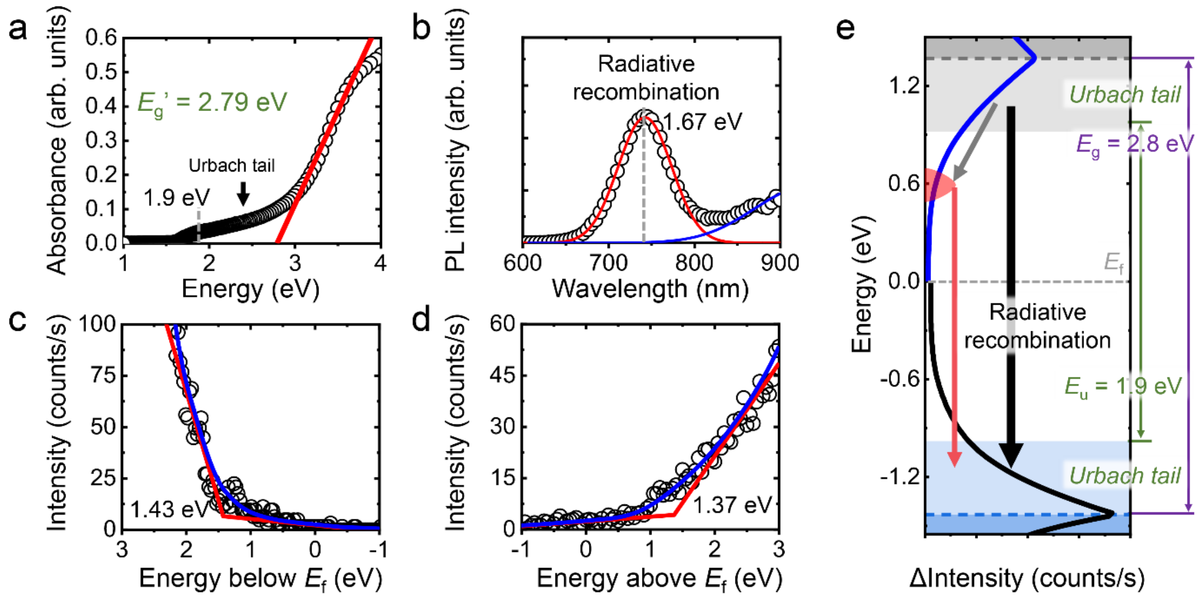

Supplementary Fig. 16. DOSs distribution of HF-treated InP QDs. a light absorption, **b** PL emission spectra, **c** UPS, and **d** IPES of HF-treated InP QDs. **e** Band structure of HF-treated InP QDs calculated from **a**-**d**.

To verify the mid-gap trap state, we treated the InP QDs using HF. During the treatment, P dangling bond on the surface of InP QDs is effectively passivated[40,41]. Compared to the NH_4SCN-passivated InP QDs, HF-treated QDs show negligible mid-gap states near the conduction band edge which indicates that imperfect surface passivation occurs in the mid-gap trap in InP QDs. Due to the decrease in the mid-gap trap, radiative recombination is facilitated.

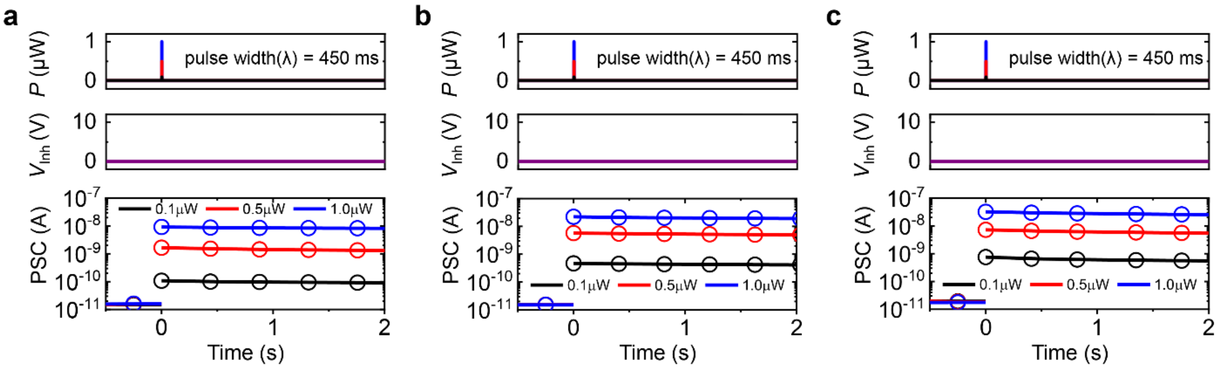

Supplementary Fig. 17. EPSC behavior of the QEPS under application of various V_{Inh} pulses. a 50mg/ml of InP QD solution was coated, **b** 25mg/ml of InP QD solution was coated, and **c** HF-treated InP QD solution was coated on the substrate, corresponding to high, intermediate, and low mid-gap trap density, respectively.

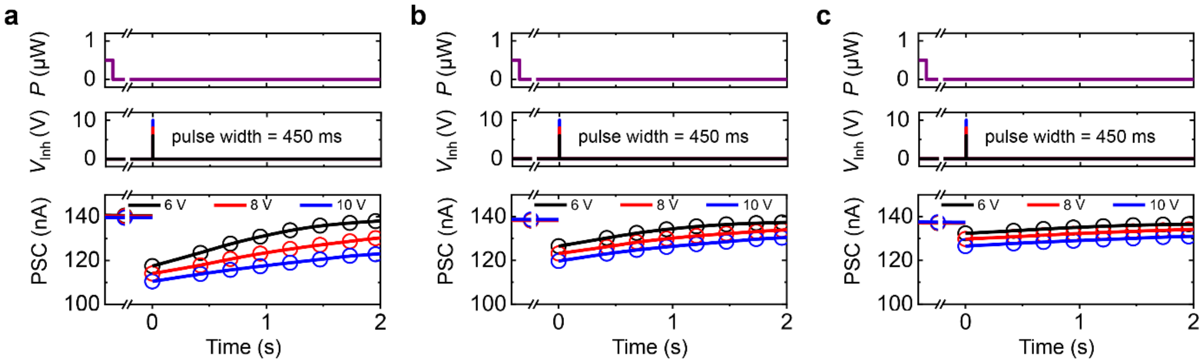

Supplementary Fig. 18. IPSC behavior of the QEPS under application of various V_{Inh} pulses. a 50mg/ml of InP QD solution was coated, **b** 25mg/ml of InP QD solution was coated, and **c** HF-treated InP QD solution was coated on the substrate, corresponding to high, intermediate, and low mid-gap trap density, respectively.

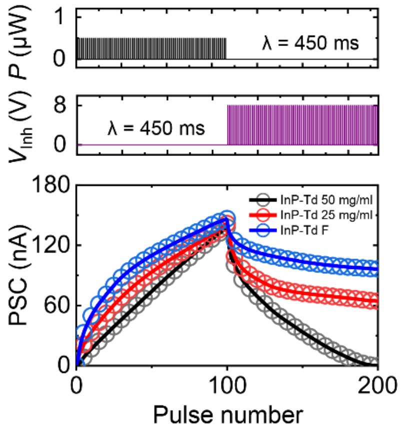

Supplementary Fig. 19. LTP/D behavior of mid-gap trap engineered QEPS. The mid-gap trap facilitates returning the increased PSC to its original state.

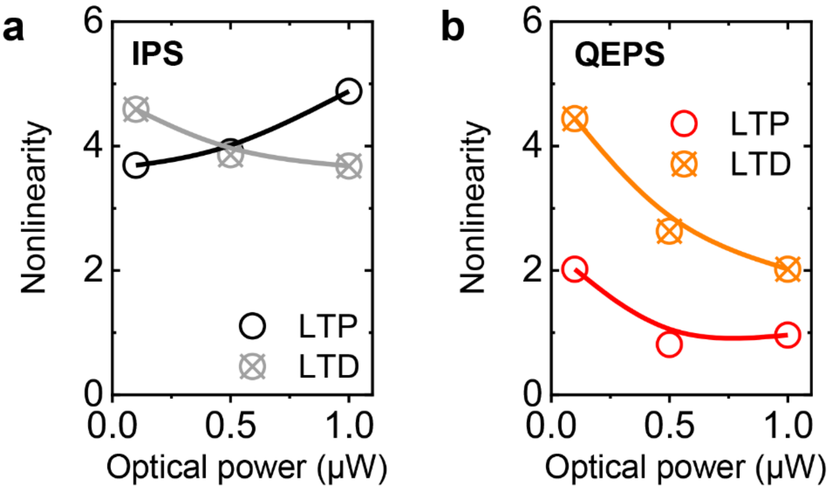

Supplementary Fig. 20. Plots of nonlinearity as a function of the optical power. Through the comparison of **a** IPS and **b** QEPS, linear LTP/D characteristics were observed due to the suppressed trap generation by mid-gap trap under the continuous V_{Inh} pulse train.

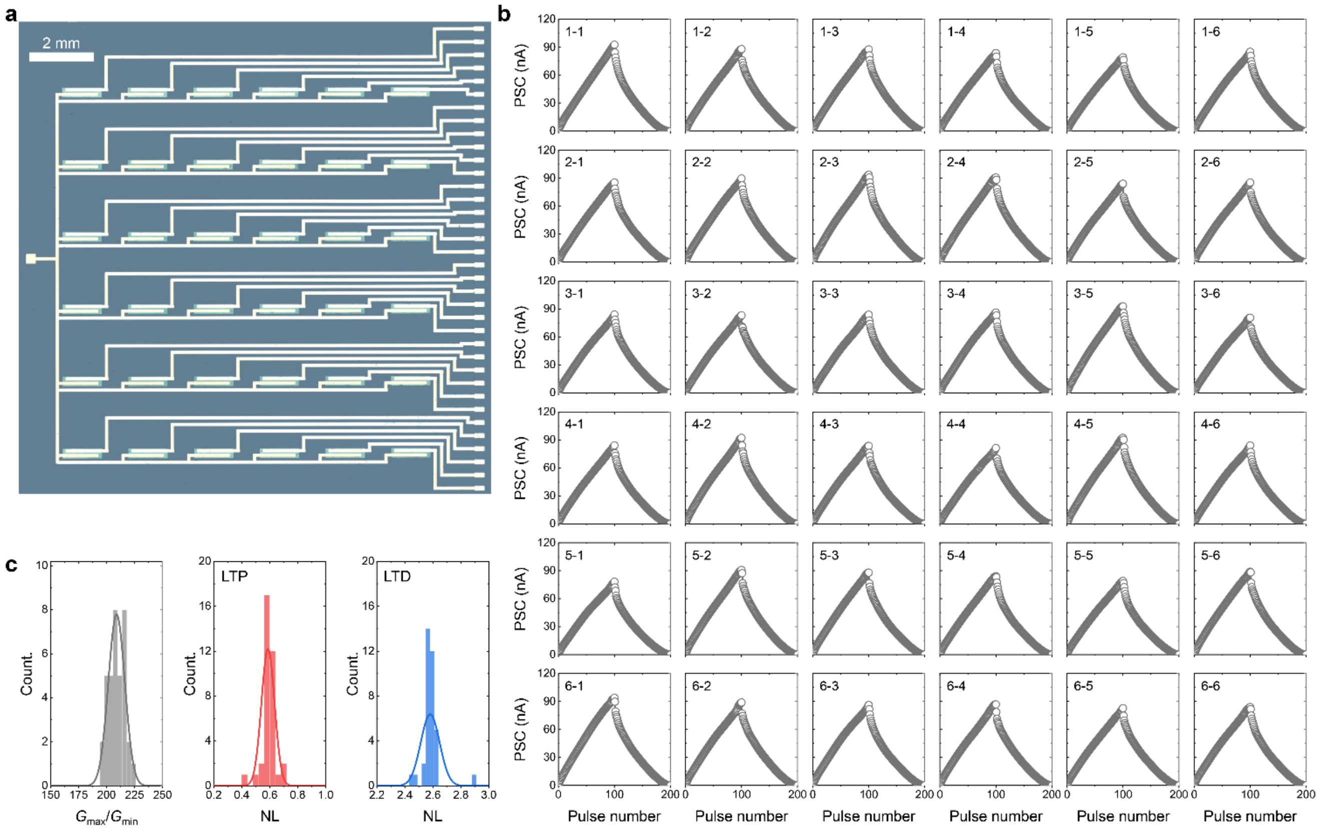

Supplementary Fig. 21. Film uniformity for 36 QEPS. **a** Optical image of a 6×6 QEPS array. **b** LTP/D behavior of each QEPS. **c** Summary of the synaptic properties for 36 QEPS.

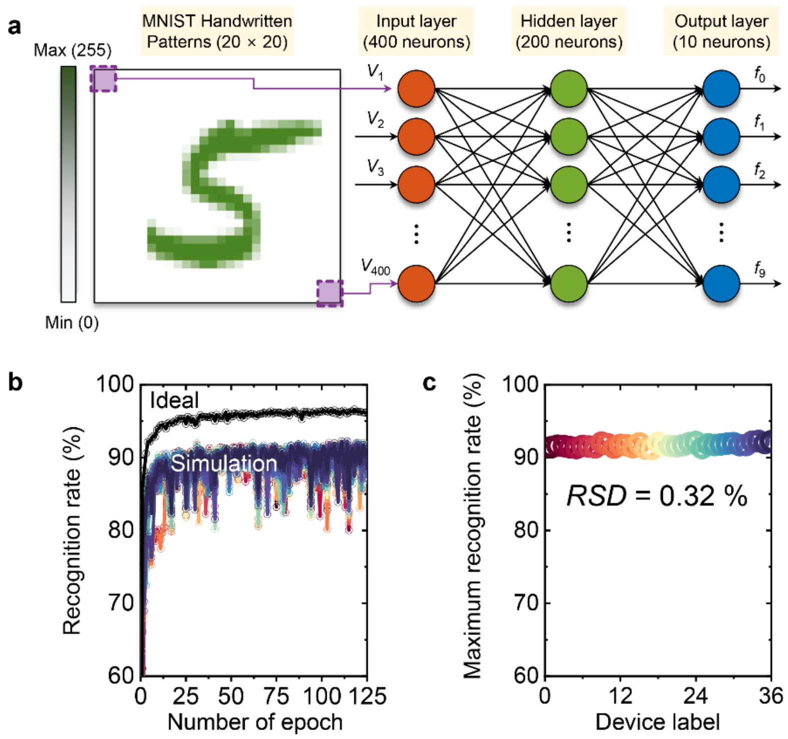

Supplementary Fig. 22. Training/recognition tasks and plot of recognition rate for MNIST digit patterns. **a** Schematic illustration of two-layer perceptron-based ANN. **b** Recognition rate as a function of number of training epochs for 36 QEPS. **c** Maximum recognition rates for 36 QEPS with a root standard deviation (RSD) of 0.32 %.

Each layer of the two-layer perceptron-based ANN consists of 400, 200, and 10 neurons for the input layer, hidden layer, and output layer, respectively. Each epoch for the recognition rate consists of 8,000 training numbers.

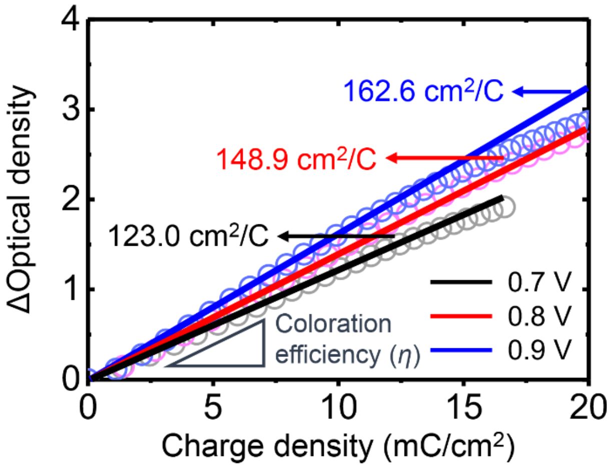

Supplementary Fig. 23. Plot of optical density difference as a function of the injected charge density. The coloration efficiency (η) of the EC platform is evaluated through the slope under application of various voltages.

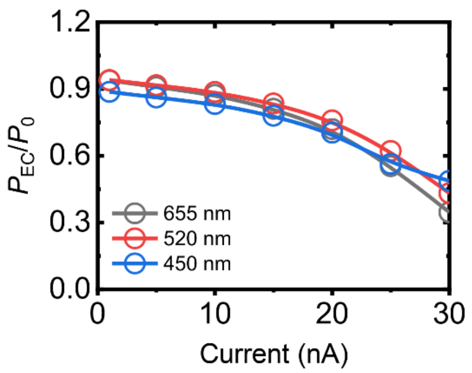

Supplementary Fig. 24. Plot of P_{EC}/P_0 versus current, showing the light intensity modulation capability of the EC device at various wavelengths.

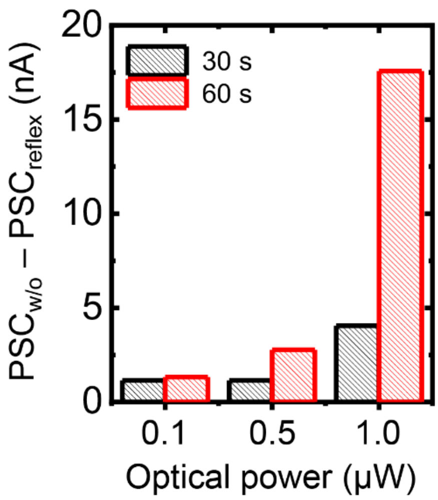

Supplementary Fig. 25. Plot of $PSC_{w/o}$ - PSC_{reflex} according to the optical power of the light input. The large PSC difference at a higher optical power was observed due to the autonomous coloration of EC device.

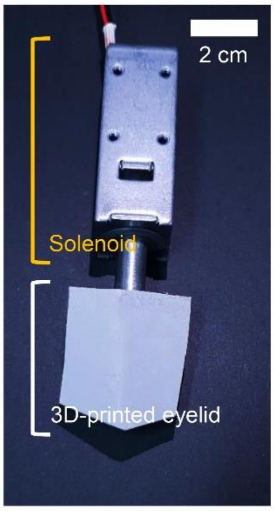

Supplementary Fig. 26. Photographic image of the S-eyelid. The 3D-printed eyelid was connected to the edge of the solenoid**.**

Supplementary Table 1. Performance characteristics of EC device.

The maximum transmittance contrast (ΔT_{max}) was measured from Figure 3e. The response time, defined as the period to achieve a 90% change in ΔT_{max}, is extracted from the time-dependent transmittance profile during coloring ($\Delta t_{c,90\%}$) and self-bleaching processes ($\Delta t_{b,open,90\%}$). The coloration efficiency (η) of the EC platform is evaluated through the slope of **Supplementary Fig. 23**.

Applied voltage (V)	ΔT_{max} (%)	$\Delta t_{c,90\%}$ (s)	$\Delta t_{b,open,90\%}$ (s)	η (cm^2/C)
0.7	~ 96.2	~ 37	~ 85	~ 123.0
0.8	~ 98.5	~ 15	~ 114	~ 148.9
0.9	~ 99.8	~ 8	~ 120	~ 162.6
